# Myogenic tissue nanotransfection improves muscle torque recovery following volumetric muscle loss

**DOI:** 10.1038/s41536-022-00259-y

**Published:** 2022-10-20

**Authors:** Andrew Clark, Subhadip Ghatak, Poornachander Reddy Guda, Mohamed S. El Masry, Yi Xuan, Amy Y. Sato, Teresita Bellido, Chandan K. Sen

**Affiliations:** 1 Indiana Center for Regenerative Medicine and Engineering, Indiana University Health Comprehensive Wound Center, Department of Surgery, Indiana University School of Medicine, Indianapolis, IN 46202 USA; 2 Birck Nanotechnology Center and Weldon School of Biomedical Engineering, Purdue University, West Lafayette, IN 47907 USA; 3 Department of Anatomy and Cell Biology, Indiana University School of Medicine, Indianapolis, IN 46202 USA; 4 Central Arkansas Veterans Healthcare System, Little Rock, AR 72205 USA; 5 Division of Endocrinology, Department of Medicine, Indiana University School of Medicine, Indianapolis, IN 46202 USA; 6 Indiana Center for Musculoskeletal Health, Indianapolis, IN 46202 USA; 7 Richard L. Roudebush Veterans Administration Medical Center, Indianapolis, IN 46202 USA; 8 Present Address: Department of Physiology and Biophysics, University of Arkansas for Medical Sciences, Little Rock, AR 72205 USA

**Keywords:** Gene therapy, Skeletal muscle

## Abstract

This work rests on our non-viral tissue nanotransfection (TNT) platform to deliver *MyoD* (TNT_MyoD_) to injured tissue in vivo. TNT_MyoD_ was performed on skin and successfully induced expression of myogenic factors. TNT_MyoD_ was then used as a therapy 7 days following volumetric muscle loss (VML) of rat *tibialis anterior* and rescued muscle function. TNT_MyoD_ is promising as VML intervention.

Volumetric muscle loss (VML) is the traumatic or surgical loss of skeletal muscle that results in chronic muscle weakness^1^. The functional deficits are often much larger than the proportion of muscle mass lost^2^. In preclinical models, a 20% loss in mass can result up to a 90% functional deficit^3^. Incapable of regenerating the amount of lost tissue, the VML defect region fills with a fibrotic scar containing an exorbitant amount of fibroblasts and immune cells^4^. Current clinical treatments for VML are physical therapy or autologous tissue transfer^5^. These treatments experience comorbidity and thus effort has been placed to find new treatments. Work using acellular matrices alone has failed to create de novo muscle fibers >0.5 mm into the defect region^5^, and thus the most successful approaches of restoring function have utilized some type of cell transplant^6^. This research work tests tissue nanotransfection (TNT) based gene therapy as an acellular treatment for VML, with the goal of overexpressing *MyoD* to affect cells in the fibrotic region towards improved functional rescue of the affected muscle. Fibroblasts, abundant in the fibrotic defect, are known for their inherent plasticity and are known to readily convert into myogenic cells in response to *MyoD* delivery^7^. To eliminate risks associated with viral gene delivery^8^, the electrophoretic TNT approach was adopted.

We have previously reported a non-viral TNT technology approach to deliver plasmids and achieve direct cell reprogramming in vivo in the skin^9–11^. Unlike plasmid delivery *via* direct injection that is stochastic and highly localized in the tissue, TNT approach employs nanochannel-based deterministic gene delivery optimized to achieve in vivo tissue reprogramming^12–17^. The current work employs second-generation TNT silicon chip (TNT_2.0_), the nanofabrication of which has been recently published^17^.

The TNT_2.0_ device hardware was modified to include needles, which allow for better contact with the transfected tissue and variable penetration depth of plasmids into the targeted tissue depending on applied voltage (Fig. 1a–d)^17^. The hypothesis that TNT_MyoD_ can rescue muscle function following VML was tested. To investigate the potential for *MyoD*-mediated in vivo conversion of mesodermal cells into myogenic cells, TNT was performed with plasmids encoding *MyoD* with eGFP reporter into the dorsal skin of C57Bl/6 mice (Fig. 1c, d). The transfected skin collected 24 h post-TNT_MyoD_ demonstrated eGFP expression throughout the epidermis and dermis and was absent in skin transfected with an empty vector (mock) plasmid, demonstrating successful expression of the *MyoD*-encoded plasmid in the skin (Fig. 1e). Quantification of gene expression demonstrated a transient increase abundance of *MyoD* transcript in the skin subjected to TNT_MyoD_ (Fig. 1f). In another cohort, skin tissue was harvested 10 days following TNT and immunofluorescent staining for MF20 (antibody binding all skeletal muscle myosin heavy chain isoforms) was performed. Expression of MF20 was observed in the TNT_MyoD_ group (Fig. 1g). Additionally, positive MF20 signal was accompanied by a peripheral flattened nucleus, typical of mature muscle fibers. Analysis of gene expression of transfected tissue showed an upregulation of several myogenic genes (Fig. 1h).

**Fig. 1:**
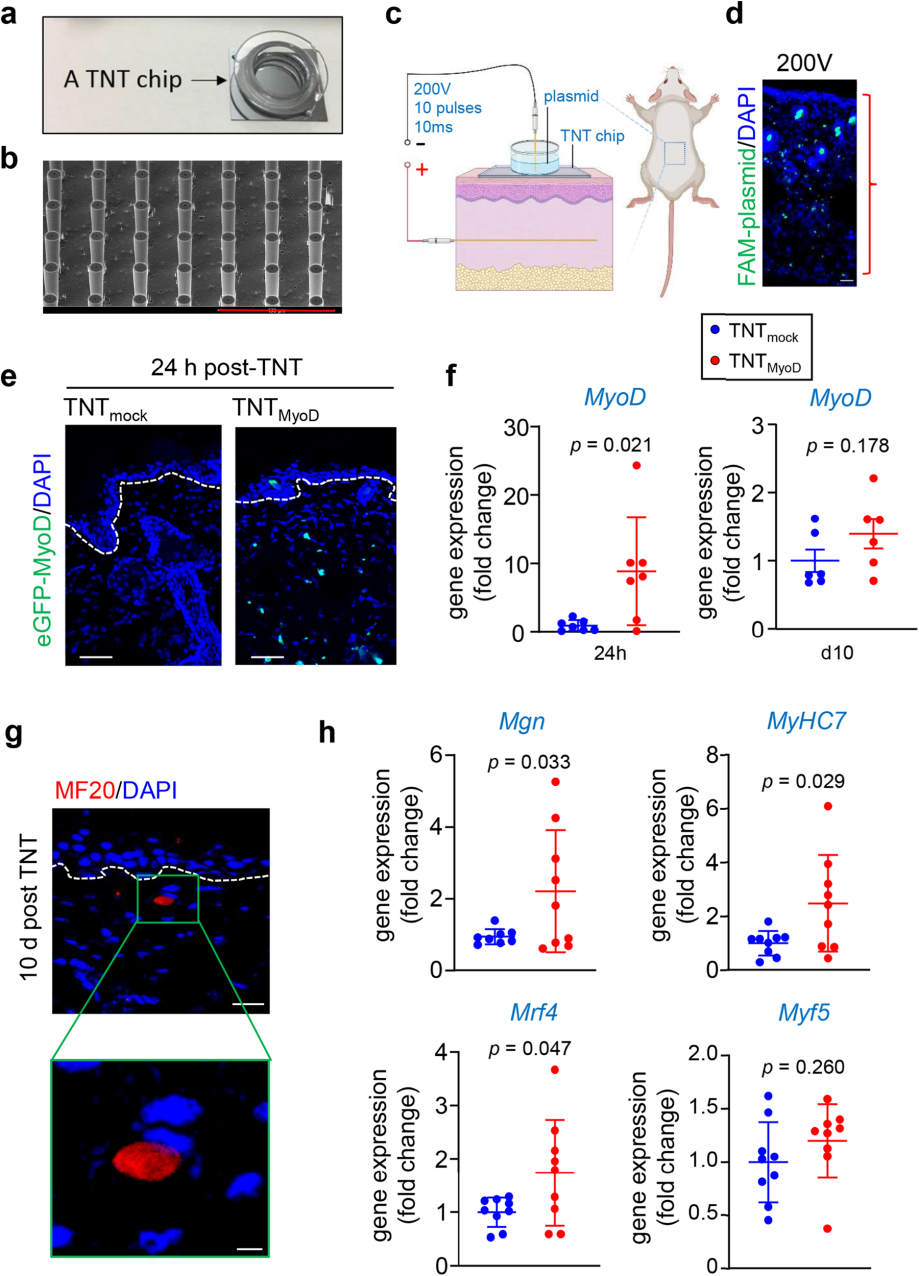
TNT_MyoD_ transfects deep into the dermis and causes myogenic reprogramming. **a** Image of TNT_2.0_ device. **b** SEM images of TNT device showing needle array projections. Scale, 400 µm. **c** Schematic diagram showing TNT set up for skin. **d** Distribution of FAM-labeled plasmids immediately after TNT using various voltages on mouse dorsal skin. Scale bar = 40 µm. **e** Visualization of eGFP signal 24 h after TNT with the eGFP-MyoD plasmid, showing expression of eGFP in epidermis and dermis. No eGFP fluorescence was detected in skin transfected with mock plasmids. The white dashed line indicates the dermal-epidermal junction. Scale, 50 µm. **f** *MyoD* expression in skin 24 h and day 10 post-TNT (*n* = 7,6). **g** Immunofluorescence staining of MF20 (myosin heavy chain) at 10 days post-TNT in the dermis. The white dashed line indicates the dermal-epidermal junction. The sections were co-stained with DAPI. Scale bar = 20 µm, 5 µm (**h**) transcript abundance of myogenic genes compared to mock-transfected skin (*n* = 9). All data are expressed as mean ± SD. Data analyzed by Student’s *t*-test. Figure **c** was created with BioRender.com.

With the success of TNT_MyoD_ inducing myogenic factor expression in the skin, its efficacy as an intervention for VML was tested. To develop a VML defect, a 6 mm full-thickness punch biopsy was taken out of the middle third of the right *tibialis anterior* muscles in Lewis rats. During the injury surgery, an in situ forming hydrogel was cross-linked in the defect to serve as a scaffold for infiltrating cells and preventing contracture^10,18^. This hydrogel consisted of 50% reduced growth factor Cultrex basement membrane extract, 20 mg/mL fibrinogen, and cross-linked with 4 U/mL of thrombin. Additionally, 10 mg of suramin per rat was dissolved in each hydrogel to act as a long-lasting inhibitor of TGF-β. TGF-β is a known inhibitor of myogenic differentiation^19^. At 7 days post-VML injury, the injured muscle was exposed and VML defect region and surrounding muscle underwent either TNT_MyoD_ or TNT_mock_ (Fig. 2a). At this 7-daytime point, acute inflammation and edema subsided and cells were able to infiltrate into the defect region. Determination of gene expression 24 h after TNT showed that muscles that underwent TNT_MyoD_ had significantly higher levels of MyoD than TNT_mock_ (Fig. 2b) that persisted till day 5 (Supplementary Fig. 1).

**Fig. 2:**
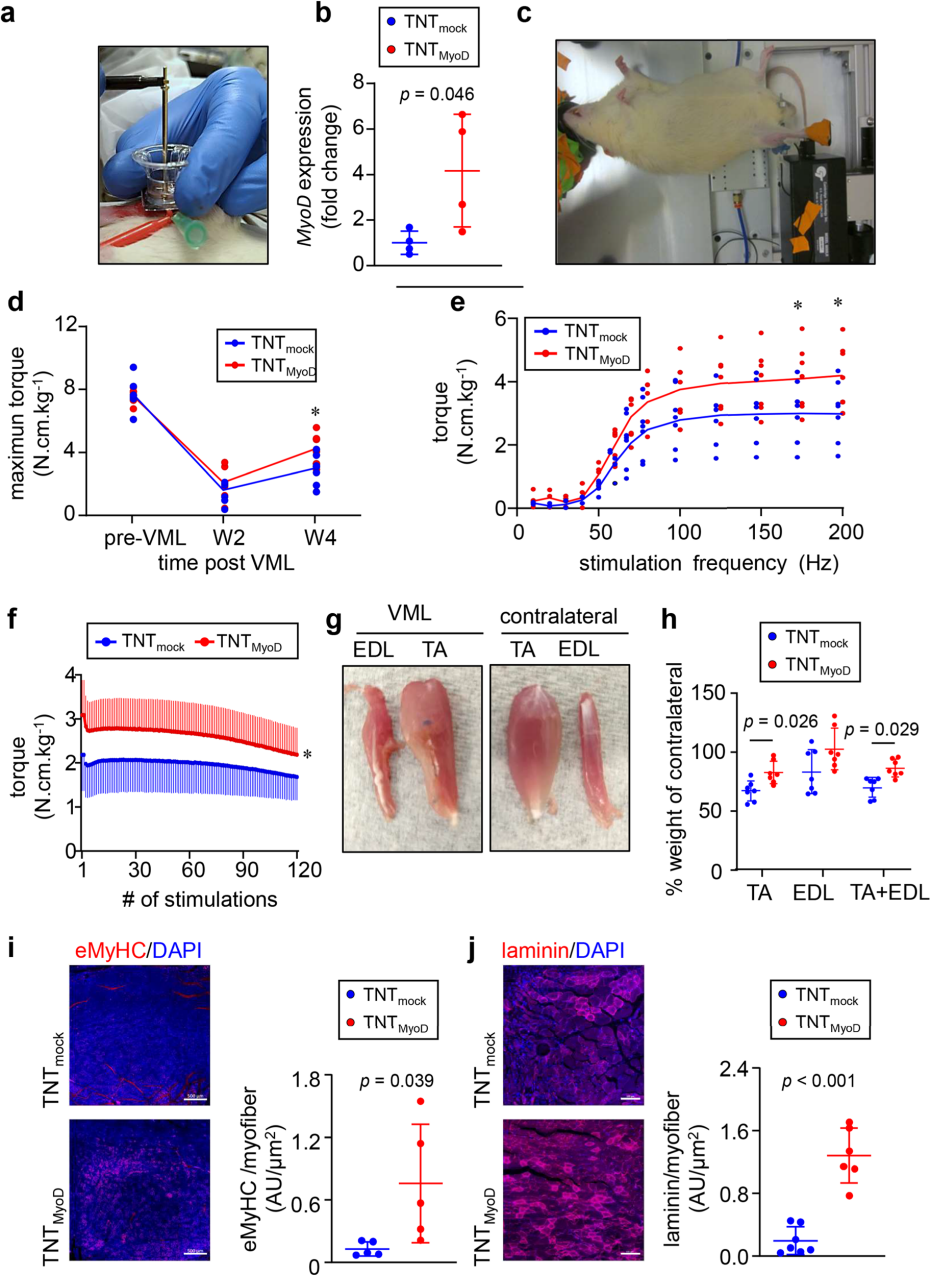
TNT_MyoD_ improves torque recovery in volumetric muscle loss. **a** Image of TNT being performed on *tibialis anterior* muscle 1 week after VML injury. **b** Gene expression of *MyoD* in VML injured muscle 24 h post-TNT (*n* = 4). **c** Image of rat undergoing muscle functional testing. **d** Maximum dorsiflexion torque produced by VML affected limb. **e** Dorsiflexion torque produced at different frequencies of direct *tibialis anterior* stimulation 4 weeks after VML(*n* = 7). **f** At 4 weeks after VML injury, dorsiflexion torque is produced after repeated electrical stimulation of the *tibialis anterior* muscle at 70 Hz. Stimulations occurred every 3 s (*n* = 7). Regression line equations for data between 60–120 stimulations: 7 = −0.0056*x + 2.387 (TNT_mock_) and *y* = −0.0086*x + 3.2345 (TNT_MyoD_). **g** Images of VML affected *tibilais anterior* and synergist *extensor digitorium longus* and their contralateral counterparts 4 weeks post-injury (3 weeks after TNT). **h** Quantification of muscle weights 4 weeks after VML (*n* = 7). **i** Immunohistochemistry of eMyHC and quantification per area of regenerating myofiber at the injury site post-TNT with either mock or *MyoD* plasmid. Scale, 500 µm (*n* = 5). **j** Immunohistochemistry of laminin and quantification per area of regenerating myofiber at the injury site post-TNT with either mock of MyoD. Scale, 100 µm (*n* = 7,6). Data are expressed as mean ± SD in **b**, **h**, and **i**. *p < 0.05. Data expressed as individual data points with mean trend lines in **d** and **e**. Data in **b**, **i**, and **j** were analyzed by Student’s *t*-test. Data in **d**, **e**, **f**, and **h** were analyzed by ANOVA.

In vivo functional tests (Aurora Scientific) were performed to measure maximal dorsiflexion torque (the ability for the muscle to contract) of the anterior compartment leg musculature upon direct stimulation of the *tibialis anterior* muscle at different frequencies (Fig. 2c). Testing was performed before injury and 2 and 4 weeks after injury (1 and 3 weeks after TNT). At 2 weeks post-injury, maximal torque was decreased by ~75% in both TNT_MyoD_ and TNT_mock_ groups compared to pre-VML (Fig. 2d). There was recovery of maximal torque between 2- and 4 weeks post-injury, which was greater in the TNT_MyoD_ group compared to TNT_mock_. An analysis of maximal torque at different stimulation frequencies revealed that the observed difference in torque only occurs at higher stimulation frequencies when the contraction becomes tetanic (Fig. 2e). A fatigue test was performed 4 weeks after injury in which the *tibialis anterior* muscle was repeatedly stimulated at 70 Hz every 3 s for 360 s and the torque produced by each stimulation was measured (Fig. 2f). This fatigue test showed a higher maximal torque in the TNT_MyoD_ group compared to TNT_mock_, indicating gains in muscle strength for the TNT_MyoD_ group. The increase in fatigue could also be due to more muscle fibers in the TNT_MyoD_ group that undergo fatigue, which is not expected from scar tissue. The TNT_MyoD_ group experienced a greater rate of decrease in torque production throughout the last half of the fatigue test. However, this may be due to TNT_mock_ beginning near their weakest torque while TNT_MyoD_ rescues maximal torque production, allowing for greater fatigue slopes before reaching their weakest torque. At the 4-week time point, functional improvements were noted. The affected skeletal muscles of these rats were harvested, and it was found that the weight of the injured *tibialis anterior*, compared to the contralateral muscle, was higher in rats that received TNT_MyoD_ (Fig. 2g, h). This muscular hypertrophy was also observed for the combined weight of the injured *tibialis anterior* plus *extensor digitorium longus* (synergist for the *tibialis anterior*) (Fig. 2g, h). This increase in muscle mass may be caused by a larger number of contractile elements such as de novo muscle fiber growth at the repair site as observed from the immunohistochemistry of muscle regenerative markers, which could be responsible for the increase in maximal force production (Fig. 2i, j, Supplementary Figs. 2, 3).

This work demonstrates TNT_MyoD_ intervention as a possible treatment to restore function to VML that can be performed without a cellular transplant. Functional improvements from TNT_MyoD_ might be further improved by the addition of plasmids encoding other factors or in combination with other types of therapies. One such possibility might be increasing neurogenic factors through TNT^20^ to rescue denervation resulting from VML^21^. Furthermore, this work describes the first use of TNT on tissue other than skin.

## Methods

### Animals

C57BL/6 mice (aged 8–12 weeks) were obtained from Jackson Laboratory. Lewis rats (aged 8–10 weeks) were obtained from Charles River. All animal procedures were conducted following IACUC approval (protocol # 18061 and 21168). After euthanasia, for rats that underwent VML surgery, both *tibialis anteriors* and *extensor digitorius longus* muscles were isolated, weighed, and imaged with digital photography. Rats were assigned to groups to make similar averages for all groups for both pre-VML specific force and body weight and then the groups were assigned to a treatment using a random number generator (www.random.org).

### Volumetric muscle loss surgery

Rats were anesthetized. An incision was made through the skin and fascia overlying the right tibialis anterior muscle. A full-thickness 6 mm biopsy was taken through the middle of the tibialis anterior. A solution consisting of 50% reduced growth factor Cultrex basement membrane extract (R&D Systems, 3533-010-02), 20 mg/mL fibrinogen (Sigma Aldrich, F8630), and 10 mg of suramin was prepared for each rat. In the defect, ~80 µL of the above solution was injected and cross-linked with 4 U/mL of thrombin (Sigma Aldrich, T7326). Single uninterrupted sutures (Henry Schein, 900–7479) were used to mark the superior and inferior edges of the defect region. The fascia and skin were then sutured closed^22^.

### Tissue nanotransfection (TNT) chip manufacturing

The TNT chip was manufactured as previously published^17^. In a cleanroom, hollow microneedles were fabricated on a double-side polished Silicon (Si) wafer using a standard semiconductor process. The Si wafer was wet oxidized 4 µm to form a hard mask for deep silicon etching. On one side of the Si wafer, a positive photoresist of AZ 9260 was spin-coated and prebaked at 110 °C for 10 min. A circle array (20–30 µm) was created using a direct laser writing system which was then developed in diluted AZ400K. The oxide was removed using CHF3 plasma etching followed by Bosch process deep silicon etching to create 350–450 µm in-depth reservoirs. On the other side of the Si chip, a donut-shaped pattern was exposed onto the resist and transferred to the oxide using the above steps. Bosch process was then used to etch hollow microneedles that connected to the reservoirs on the other end of the chip. Finally, a SiO_2_ thin film was coated with PECVD (plasma-enhanced chemical vapor deposition) to shrink the bore size of the microneedle to ~4 µm. SEM imaging was performed on the chip to analyze the structure^17,23^.

### Tissue nanotransfection (TNT)

#### Dorsal mouse skin

Animals were anesthetized using inhaled isoflurane. The skin to be transfected was depilated and then tape stripped 6 times followed by rubbing with exfoliating cream. Once the skin was prepared, a positive needle electrode was inserted into the skin. The TNT nanochip was plated directly on the skin above the positive electrode. A gold-plated negative electrode was placed in a solution of plasmids (*MyoD* expressing or empty vector (mock) or fam-labeled plasmids) in the reservoir above the TNT nanochip. Pulsed electrical stimulation (10 pulses, 200 V in amplitude, duration of 10 ms per pulse) was then applied across the electrodes to cause transfection of the skin^12,15,16,24^.

#### Rat tibialis anterior

TNT was performed 7 days after the VML surgery. The rat was anesthetized using inhaled isoflurane and the surgical site was sterilized with ethanol and betadine. An incision was made through the skin and fascia to expose the tibialis anterior (TA) (at the same location as the incision from the VML surgery). A needle cathode was inserted under the VML defect region of the TA. The TNT nanochip was plated directly on the muscle above the positive electrode. A gold anode was inserted in the plasmid solution containing 0.34 ug/uL of *MyoD* expressing or empty vector (mock) plasmid. A current of 200 V was passed through the TNT nanochip/muscle interface for 10 ms with 100 ms intervals for 10 pulses. Upon completion of TNT, the fascia and skin were closed. Following TNT, the delivery potential list was checked to ensure the successful delivery of plasmids.

### Delivery efficiency

To visualize penetration of plasmids immediately following TNT, the area transfected was immediately harvested and flash-frozen in OCT. Sections were cut at 16 µm. Sections were incubated in acetone for 1 min and then counter-stained with DAPI. Sections were then imaged using Zeiss Axio Scan.Z1^25,26^.

An eGFP-tagged *MyoD*-encoded plasmid, was used to localize plasmid expression in tissue following TNT. The skin transfected was collected 24 h after TNT and flash frozen in OCT. Sections were cut at 16 µm and, without thawing, incubated overnight at −20 °C in an air-tight chamber containing a paper towel soaked with 37% paraformaldehyde to fix the sections *via* formaldehyde vapor^15^. The sections were then rinsed in PBS and counter-stained with DAPI. Sections were then imaged using Zeiss LSM880 confocal microscope.

### In vivo muscle functional testing

A heat therapy pump (37 °C) was used to maintain a constant temperature for the apparatus during muscle function testing. Rats were anesthetized by continuous administration of isoflurane. The right hind limb of the rat was shaved and sterilized. The foot was secured to a foot pedal using a combination of tape and sticky tack. The foot was placed on the foot pedal at a 90-degree angle with the leg positioned perpendicular to the foot pedal. A knee clamp was then used to secure the leg. Two monopolar electrodes were inserted under the skin, one positioned medial and one lateral of the tibialis anterior to stimulate a dorsiflexion twitch response. The maximum amperage was used for stimulating muscle contractions (which we found to be 3 mA). A simulation program was run that recorded maximum isometric torque (N*m) for stimulation frequencies ranging from 10–200 Hz, occurring every 45 sec, with a pulse width of 0.2 ms and train duration of 200 ms. Upon completion of this force-frequency, 3 min of rest was given before beginning the fatigue test. During the fatigue test, rats were stimulated as described above but at 70 Hz every 3 s for a total of 120 stimulations. The animal was then removed from the apparatus and returned to its cage upon recovery from anesthesia^15^.

### Immunohistochemistry

Tissue was flash-frozen in OCT or embedded in paraffin post-fixation in 4% formalin. OCT sections were cut at 12 µm and were fixed in acetone for 1 min. Paraffin sections were cut at 7 µm. Sections were blocked in 10% normal goat serum and mouse Ig blocking reagent (Vector Labs, MKB-2213) for 1 h. MF20 (DSHB, 1:16) and eMyHC (DSHB, F1.652, 5ug/mL, 1:200), Pax 3 (DSHB, 1:100), Caveolin 1 (Abcam, ab18199, 1:50), Pax 7(DSHB, 1:100), Myf5 (Abcam, ab125301, 1:200) and Laminin (Abcam, ab11575, 1:500) primary antibodies were incubated on the sections at +4 °C overnight. Sections were then incubated with fluorescent-tagged secondary antibody Alexa 488-tagged α-mouse (1:200) or Alexa 568-tagged α-mouse (1:200) and counter-stained with DAPI. Sections were then imaged using a laser-scanning confocal system (Carl Zeiss LSM 888) and (Axio Scan.Z1, Zeiss, Germany). One section from the center of the biopsy were analysed using at least 3 ROI. Quantification of fluorescent intensity of image was analyzed using Zen software (Zen blue 3.1)^15,25,26^. All the ROI and the mean intensities were plotted graphically as individual data points.

### RNA extraction and qRT-PCR

RNA was extracted from tissue using the mirVana miRNA extraction kit (Ambion) following the manufacturer’s protocol. Reverse transcription was then performed using SuperScript™ VILO™ cDNA Synthesis Kit (Invitrogen) following the manufacturer’s protocol. The resulting cDNA was then undergone qRT-PCR on a QuantStudio 3 using SYBR green master mix (Applied Biosystems)^27^.

### Statistical analysis

Data from control vs test samples were coded for analysis in a blinded manner. Data were checked for normalcy by plotting data on a normal probability plot. After normalcy was confirmed, comparisons utilizing two groups (control vs treatment) were made using independent sample t-tests with the significance value being defined as p < 0.05. For the functional assays utilizing multiple groups, a repeated-measures ANOVA was used. This ANOVA was made with the following main effects: treatment, frequency, VML defect size, and interaction effect: treatment*frequency. Body weight was not used as a main effect for the ANOVA due to torque already being normalized to body weight. Following significance, Tukey’s post hoc test was used as a multiple comparison method. Statistical analyses were performed in JMP 14 Pro or GraphPad Prism 8. Unless otherwise mentioned, all n represents biological replicates.

### Reporting summary

Further information on research design is available in the Nature Research Reporting Summary linked to this article.

## Supplementary information

Supplementary Material

REPORTING SUMMARY

## Data Availability

The data that support the findings of this study are available from the corresponding author (C.K.S) upon request.
