# Supplementary material for: Myogenic tissue nanotransfection improves muscle torque recovery following volumetric muscle loss
Source: NPJ Regen Med. 2022 Oct 20;7:63. doi: 10.1038/s41536-022-00259-y (PMC9585072; doi:10.1038/s41536-022-00259-y)
Supplement: Supplementary file 1 — Supplementary Material [file 41536_2022_259_MOESM1_ESM.pdf]

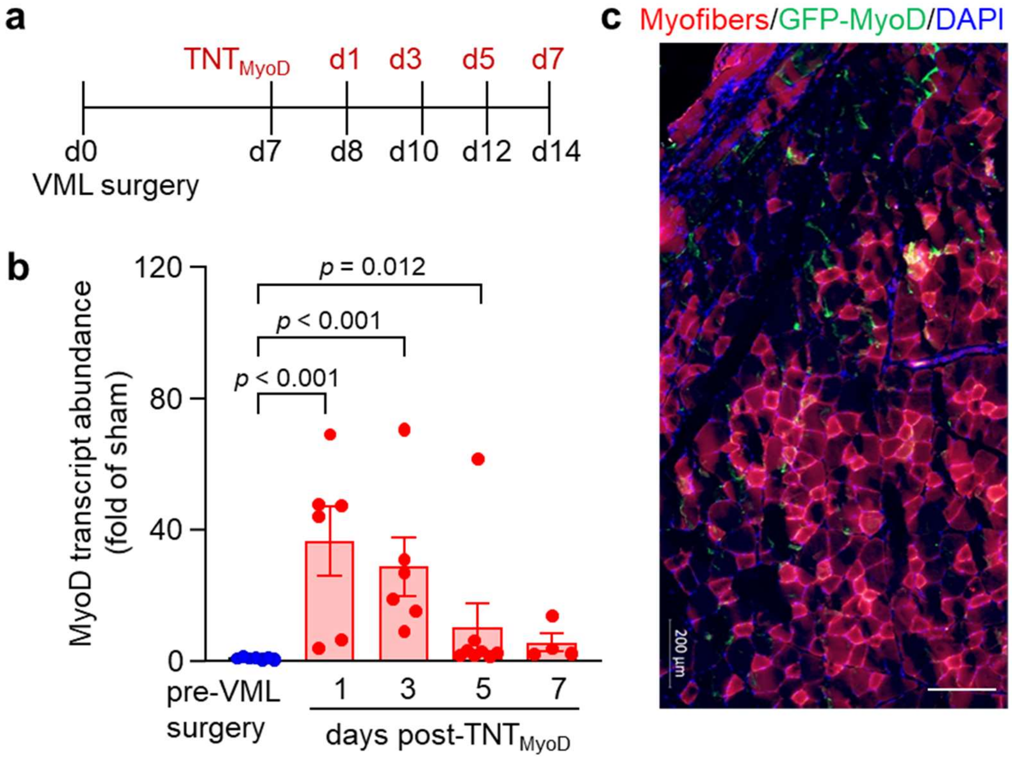

Supplementary Figure 1: (**a**) Experimental design. (**b)** Time kinetics of MyoD transcript abundance following TNT at day 7 post-VML surgery. Data were shown as mean ± SD and analysis was performed using one-way ANOVA using *post-hoc* Sidak's multiple comparison test. **(c)** Immunofluorescence staining of mouse skeletal muscle showing expression of the MyoD plasmid with GFP reporter 24h post-TNT (day 7 post-VML surgery). Scale, 200μm.

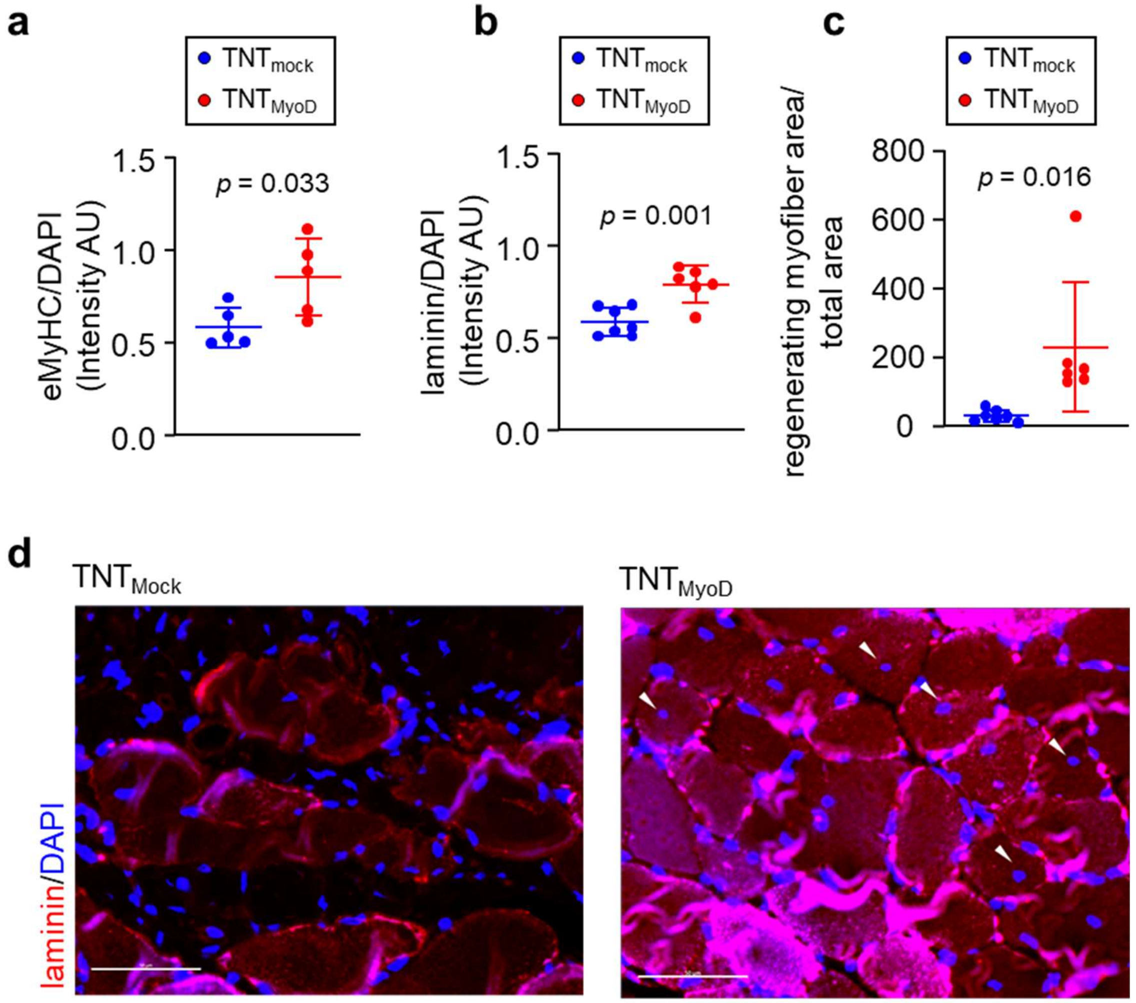

Supplementary Figure 2: (a-b) Quantification of mean intensity of eMyHC (a) and laminin (b). **(c)** Quantification of the ratio of regenerating myofiber area to the total area. (n=6). All data were shown as mean ± SD and analysis was performed using Student's t-test. (**d**) Immunohistochemistry of Laminin (red) with DAPI co-staining. Scale, 50µm. The white arrowhead shows the presence of centrally located nuclei in regenerating muscle post-TNT_{MyoD}. Related to Figure 2j

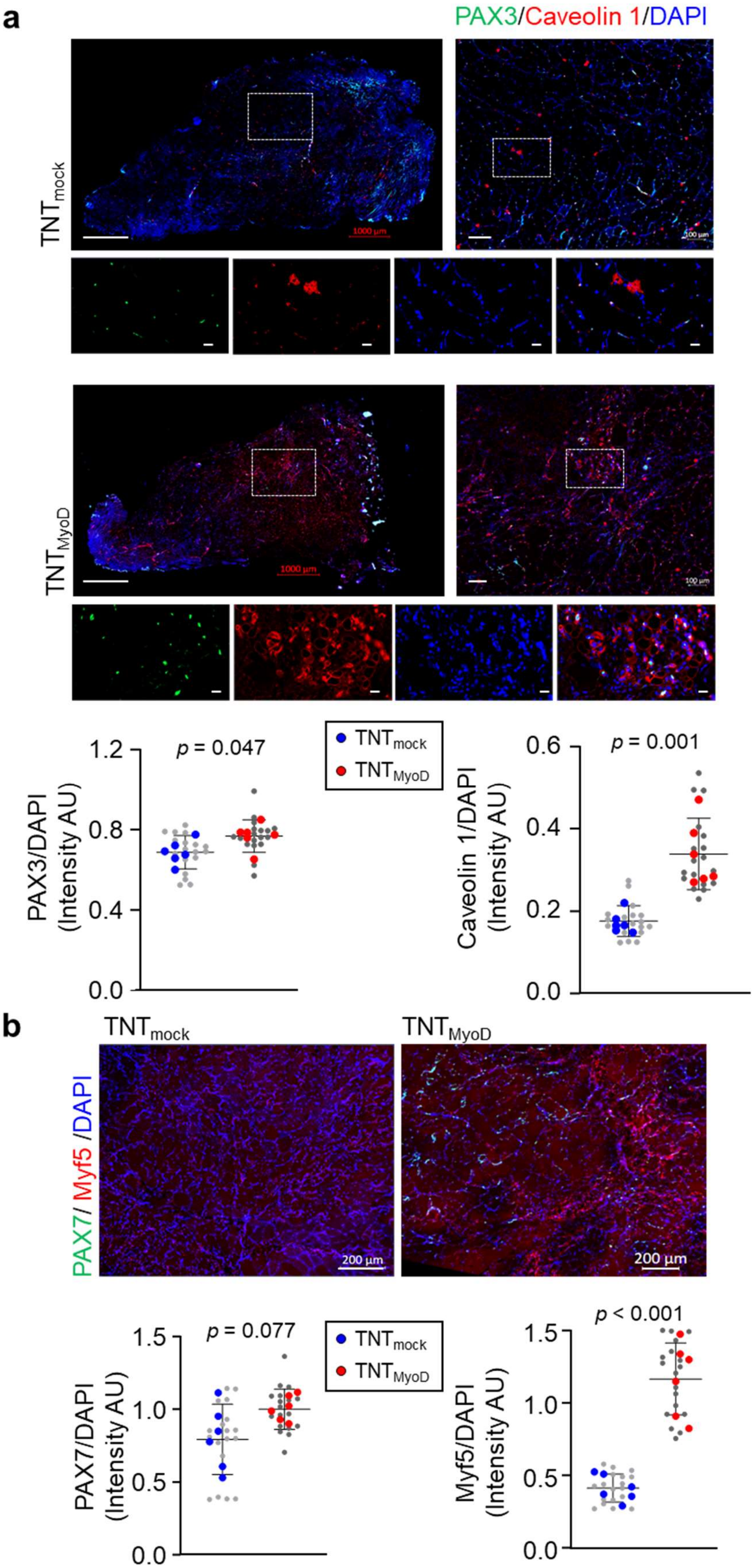
a
PAX3/Caveolin 1/DAPI
TNTmock
1000 µm
100 µm
TNTMyoD
1000 µm
100 µm
p = 0.047
TNTmock
TNTMyoD
PAX3/DAPI (Intensity AU)
1.2
0.8
0.4
0.0
p = 0.001
Caveolin 1/DAPI (Intensity AU)
0.6
0.4
0.2
0.0
b
TNTmock
TNTMyoD
PAX7/ Myf5 /DAPI
200 µm
200 µm
p = 0.077
TNTmock
TNTMyoD
PAX7/DAPI (Intensity AU)
1.5
1.0
0.5
0.0
p < 0.001
Myf5/DAPI (Intensity AU)
1.5
1.0
0.5
0.0

Supplementary Figure 3: (**a**) Immunohistochemistry and quantification of Pax3 (green) and Caveolin 1 (red) at the injury site 4 weeks post-TNT with either mock or MyoD. Scale, 1000µm, 100µm, and 20µm. (**b**) Immunohistochemistry and quantification of Pax7 (green) and Myf5 (red) at the injury site 4 weeks post-TNT with either mock or MyoD. Scale, 200µm. Each dot corresponds to one quantified ROI, except the blue and red dots, which correspond to the mean of each animal. At least 2 ROI per animal. (n=6). All data were shown as mean ± SD and analysis was performed using Student's t-test.
