# Supplementary material for: Myogenic tissue nanotransfection improves muscle torque recovery following volumetric muscle loss
Source: NPJ Regen Med. 2022 Oct 20;7:63. doi: 10.1038/s41536-022-00259-y (PMC9585072; doi:10.1038/s41536-022-00259-y)
Supplement: Supplementary file 2 — REPORTING SUMMARY [file 41536_2022_259_MOESM2_ESM.pdf]

nature portfolio

Corresponding author(s): Chandan K. Sen

Last updated by author(s): Sep 14, 2022

# Reporting Summary

Nature Portfolio wishes to improve the reproducibility of the work that we publish. This form provides structure for consistency and transparency in reporting. For further information on Nature Portfolio policies, see our Editorial Policies and the Editorial Policy Checklist.

## Statistics

For all statistical analyses, confirm that the following items are present in the figure legend, table legend, main text, or Methods section.

| n/a | Confirmed | |
|---|---|---|
| ☐ | ☒ | The exact sample size (*n*) for each experimental group/condition, given as a discrete number and unit of measurement |
| ☐ | ☒ | A statement on whether measurements were taken from distinct samples or whether the same sample was measured repeatedly |
| ☐ | ☒ | The statistical test(s) used AND whether they are one- or two-sided<br>*Only common tests should be described solely by name; describe more complex techniques in the Methods section.* |
| ☐ | ☒ | A description of all covariates tested |
| ☐ | ☒ | A description of any assumptions or corrections, such as tests of normality and adjustment for multiple comparisons |
| ☐ | ☒ | A full description of the statistical parameters including central tendency (e.g. means) or other basic estimates (e.g. regression coefficient) AND variation (e.g. standard deviation) or associated estimates of uncertainty (e.g. confidence intervals) |
| ☐ | ☒ | For null hypothesis testing, the test statistic (e.g. *F*, *t*, *r*) with confidence intervals, effect sizes, degrees of freedom and *P* value noted<br>*Give P values as exact values whenever suitable.* |
| ☒ | ☐ | For Bayesian analysis, information on the choice of priors and Markov chain Monte Carlo settings |
| ☒ | ☐ | For hierarchical and complex designs, identification of the appropriate level for tests and full reporting of outcomes |
| ☒ | ☐ | Estimates of effect sizes (e.g. Cohen's *d*, Pearson's *r*), indicating how they were calculated |

*Our web collection on statistics for biologists contains articles on many of the points above.*

## Software and code

Policy information about availability of computer code

| | |
|---|---|
| Data collection | N/A |
| Data analysis | N/A |

For manuscripts utilizing custom algorithms or software that are central to the research but not yet described in published literature, software must be made available to editors and reviewers. We strongly encourage code deposition in a community repository (e.g. GitHub). See the Nature Portfolio guidelines for submitting code & software for further information.

## Data

Policy information about availability of data

All manuscripts must include a data availability statement. This statement should provide the following information, where applicable:

- Accession codes, unique identifiers, or web links for publicly available datasets
- A description of any restrictions on data availability
- For clinical datasets or third party data, please ensure that the statement adheres to our policy

The data that support the findings of this study are available from the corresponding author upon reasonable request.

## Human research participants

Policy information about studies involving human research participants and Sex and Gender in Research.

| | |
|---|---|
| Reporting on sex and gender | N/A |
| Population characteristics | N/A |
| Recruitment | N/A |
| Ethics oversight | N/A |

Note that full information on the approval of the study protocol must also be provided in the manuscript.

# Field-specific reporting

Please select the one below that is the best fit for your research. If you are not sure, read the appropriate sections before making your selection.

☒ Life sciences ☐ Behavioural & social sciences ☐ Ecological, evolutionary & environmental sciences

For a reference copy of the document with all sections, see nature.com/documents/nr-reporting-summary-flat.pdf

# Life sciences study design

All studies must disclose on these points even when the disclosure is negative.

| | |
|---|---|
| Sample size | Immunohistochemistry, RT-qPCR, muscle function tests were performed on multiple independent biological replicates (n shown in figure legends). No statistical methods were used to predetermine sample size. For all animal studies, group sizes are reported in the main text, figure legend or within the figure. We used group sizes sufficient to detect statistically reliable effects. |
| Data exclusions | Data were excluded from experiments only if animals died or only when TNT delivery was not up to the standard. |
| Replication | All experimental findings reported here were successfully replicated across multiple biological samples (n reported in each figure legend). All immunofluorescence was performed on a minimum 3 independent skin or wound-edge tissue samples. |
| Randomization | Animals were randomly assigned using a computer based algorithm (www.random.org). |
| Blinding | Samples were coded and data analysis were performed by individual who was blinded to the origin of the samples. All analyses were performed in an automated manner across conditions. |

# Reporting for specific materials, systems and methods

We require information from authors about some types of materials, experimental systems and methods used in many studies. Here, indicate whether each material, system or method listed is relevant to your study. If you are not sure if a list item applies to your research, read the appropriate section before selecting a response.

## Materials & experimental systems

| n/a | Involved in the study | |
|---|---|---|
| ☐ | ☒ | Antibodies |
| ☒ | ☐ | Eukaryotic cell lines |
| ☒ | ☐ | Palaeontology and archaeology |
| ☐ | ☒ | Animals and other organisms |
| ☒ | ☐ | Clinical data |
| ☒ | ☐ | Dual use research of concern |

## Methods

| n/a | Involved in the study | |
|---|---|---|
| ☒ | ☐ | ChIP-seq |
| ☒ | ☐ | Flow cytometry |
| ☒ | ☐ | MRI-based neuroimaging |

## Antibodies

| | |
|---|---|
| Antibodies used | 1. MF20 (DSHB, 1:16)<br>2. eMyHC (DSHB, F1.652, 5ug/mL, 1:200)<br>3. Pax 3 (DSHB, 1:100)<br>4. Caveolin 1 (Abcam, ab18199, 1:50) |

| | |
|---|---|
| | 5. Pax 7 (DSHB, 1:100)<br>6. Myf5 (Abcam, ab125301, 1:200)<br>7. Laminin (Abcam, ab11575, 1:500) |
| Validation | All antibodies used are commercially available and validated by the vendor for the assay and species used in the study. Specific validation information for each antibody is available on the vendor's website. Additional validation for immunohistochemistry was performed using isotype controls. |

## Animals and other research organisms

Policy information about studies involving animals; ARRIVE guidelines recommended for reporting animal research, and Sex and Gender in Research

| | |
|---|---|
| Laboratory animals | 1. C57BL/6 mice (aged 8-12 weeks) were obtained from Jackson Laboratory.<br>2. Lewis rats (aged 8-10 weeks) were obtained from Charles River. |
| Wild animals | Study did not involve wild animals |
| Reporting on sex | Both sexes were used |
| Field-collected samples | Study did not involve samples collected in the field. |
| Ethics oversight | All animal studies were performed in accordance with IACUC approved at the Laboratory Animal Resource Center, Indiana University. |

Note that full information on the approval of the study protocol must also be provided in the manuscript.
